# Attenuated alpha oscillation and hyperresponsiveness reveals impaired perceptual learning in migraineurs

**DOI:** 10.1186/s10194-022-01410-2

**Published:** 2022-04-05

**Authors:** Chun Yuen Fong, Wai Him Crystal Law, Johannes Jacobus Fahrenfort, Jason J. Braithwaite, Ali Mazaheri

**Affiliations:** 1 Center for Evolutionary Cognitive Sciences, Graduate School of Art and Sciences, University of Tokyo, Meguro-ku, Tokyo, 153-8902 Japan; 2 International College of Liberal Arts (iCLA), Yamanashi Gakuin University, 2-7-17 Sakaori, Kofu City, Yamanashi, 400-0805 Japan; 3 Department of Psychology, University of Amsterdam, Amsterdam, 1001 NK the Netherlands; 4 Amsterdam Brain and Cognition (ABC), University of Amsterdam, Amsterdam, 1001 NK the Netherlands; 5 Institute for Brain and Behaviour Amsterdam, Vrije Universiteit Amsterdam, Amsterdam, the Netherlands; 6 Department of Experimental and Applied Psychology - Cognitive Psychology, Vrije Universiteit Amsterdam, Amsterdam, the Netherlands; 7 Department of Psychology, Lancaster University, Lancaster, LA1 4YF UK; 8 School of Psychology, University of Birmingham, Birmingham, B15 2TT UK; 9 Centre of Human Brain Health, University of Birmingham, Birmingham, B15 2TT UK

**Keywords:** Migraine, Pattern glare, Alpha, Hyperexcitability, Perceptual learning

## Abstract

**Background:**

Anomalous phantom visual perceptions coupled to an aversion and discomfort to some visual patterns (especially grating in mid-range spatial frequency) have been associated with the hyperresponsiveness in migraine patients. Previous literature has found fluctuations of alpha oscillation (8-14 Hz) over the visual cortex to be associated with the gating of the visual stream. In the current study, we examined whether alpha activity was differentially modulated in migraineurs in anticipation of an upcoming stimulus as well as post-stimulus periods.

**Methods:**

We used EEG to examine the brain activity in a group of 28 migraineurs (17 with aura /11 without) and 29 non-migraineurs and compared their alpha power in the pre/post-stimulus period relative to the onset of stripped gratings.

**Results:**

Overall, we found that migraineurs had significantly less alpha power prior to the onset of the stimulus relative to controls. Moreover, migraineurs had significantly greater post-stimulus alpha suppression (i.e event-related desynchronization) induced by the grating in 3 cycles per degree at the 2nd half of the experiment.

**Conclusions:**

These findings, taken together, provide strong support for the presence of the hyperresponsiveness of the visual cortex of migraine sufferers. We speculate that it could be the consequence of impaired perceptual learning driven by the dysfunction of GABAergic inhibitory mechanism.

**Supplementary Information:**

The online version contains supplementary material available at 10.1186/s10194-022-01410-2.

## Introduction

Patients with migraine are known to be vulnerable to intense visual stimuli such as environmental light and grating patterns interictally [1–5]. In psychophysical experiments, migraine sufferers demonstrated a significantly lower phosphene induction threshold when their visual cortex was stimulated by transcranial magnetic stimulation (TMS) [5–8]. They were also found to be less influenced by the metacontrast masking effect [2] as well as having a higher predisposition to experience visual discomfort by viewing striped grating at spatial frequency around 2 to 4 cycles per degree (cpd) [9–11]. Some researchers have suggested that this hyperresponsiveness could be due to a disrupted GABAergic interneuron network which weakens the suppressive function of the visual cortex [1, 12, 13].

Unlike the healthy population, migraine patients have been observed to not demonstrate a reduction of visual evoked potentials (VEP) by repetitive visual stimulations, which is also known as habituation deficit [14–16]. The neural habituation can be part of a perceptual learning mechanism and may prevent excessive neuronal stress generated at sensory cortex [17, 18]. Interestingly, the habituation deficit of migraine patients was also observed in other sensory modalities [16, 19]. Whether habituation deficits directly indicate cortical hyperexcitability in migraine pathology or are associated with their abnormal visual sensations during headache-free period remains controversial. Systematic review studies have reported normal or even attenuated VEPs for migraineurs which is not consistent with the cortical hyperexcitability hypothesis [20–22]. Amongst those empirical studies in which enhanced visual evoked potentials (VEPs) for migraineurs were reported, electrophysiological responses of the initial stimulations were not always compared with the latter stimulations. Moreover, there is considerable variability in the visual stimuli used (e.g. flash-evoked, pattern-reversed-evoked, static grating) with the psychophysical properties of the stimuli not being consistent [3, 23–26].

Contradictory findings have also been observed in investigations focusing on the oscillatory activity of the electroencephalogram (EEG) of migraine patients. The majority of the previous literature looking for aberrant patterns of oscillatory activity in migraine patients has primarily focused on task-free resting-state EEG [27]. For example, one study observed migraine patients to have increased theta, delta [28] and alpha [29] resting-state activity interictally while in another study, they appeared to show reduced resting theta, alpha, beta power [30]. Recently, the spatial coherence (functional connectivity) of different frequency bands on migraine patients was also explored [31]. In addition to resting state, sensory evoked alpha rhythm of migraine sufferers had a lower coherence compared to headache-free controls.

To the best of our knowledge, there have been few, if any studies systematically looking at stimulus induced oscillatory changes in the EEG activity of migraine patients. In the current study, we focused on the oscillatory changes in the EEG as index of the cortical responsiveness of migraine patients in anticipation, as well as during the processing of the visual gratings. Our rationale for using visual stimulation was in-part motivated by previous work that found early VEP components (e.g. N75, P100, and N145) in migraine sufferers to be enhanced relative to migraine-free controls [3, 23–25]. These enhancements were speculated to result from the lack of inhibitory control over the cortical pyramidal cells during visual stimulation [32].

We examined the brain activity in a group of 28 migraineurs (17 with aura/11 without) and 29 non-migraineurs and compared the modulations of alpha power (8 – 12 Hz) induced by striped patterns of low, medium and high spatial frequencies (i.e. 0.5, 3, and 13 cpd). Visual gratings at these three frequency bands had been previously used in a behavioural task known as pattern-glare test and found to trigger different types and levels of visual experiences, with the 3 cpd being the most discomforting to migraine sufferers [33].

We focused on the period in anticipation of a visual stimulus (i.e. post-cue to pre-stimulus) as well as post-stimulus modulations of alpha activity for the different stimuli. The alpha rhythm is the prominent (often visible in recordings with the naked eye) ongoing activity found in the EEG of wakeful participants. Alpha activity is often largest in amplitude over occipital electrodes and the prevalent hypothesis is that it captures the excitability of the visual cortex and gates sensory processing [34–36]. Specifically, alpha power can facilitate the processing of a sensory input through inhibiting sensory processing in a region when power is high [37, 38]. The pre-stimulus level of alpha activity allowed us to gauge the baseline excitability of the visual cortex expecting the arrival of an upcoming stimulus whereas the post-stimulus alpha modulation gave us an insight into the resources allocated to the processing of the visual stimuli. Previous studies also found that the anticipation of more painful/discomforting stimuli were associated with greater intensity of alpha suppression [39, 40]. 

## Methods

### Participants

Our experiment included 28 self-reported female migraineurs (mean age = 20.9) and 29 healthy female controls (mean age = 19.4, age range = 18 - 30) with normal/corrected to normal visual acuity (20/25 or better). The participants as well as their data were all part of a previously published study focusing on the modulations of evoked responses [3]. All healthy participants reported no history of migraine nor any neurological and psychiatric conditions (with 5 of them reporting that one of their parents had a history of migraine). Amongst the 28 migraine patients, 17 of them were categorised as migraine with aura and 11 as migraine without aura according to the criteria of the International Headache Society (see Supplementary Table S1 and S2 for the sample characteristics for both groups) [41]. The migraine patients were not regularly taking any prophylactic medications (and had not taken any within 2 weeks of the experiment), nor had they had chronic migraine, motor migraine aura symptoms or any other comorbid neurological or psychiatric conditions. The EEG sessions were taken at the interictal period of the migraineurs (no migraine attack 1 week before and at least 2 weeks after the recordings).

### Stimuli, apparatus and trial sequences

Achromatic gratings with a Michelson contrast of 0.70 (cd/m^2^) in three different spatial frequencies (0.5, 3 and 13 cpd, also named as LF, MF, and HF) were used as visual stimuli in the current study. The stimuli were presented at the centre of a 20-inch Dell P2210 LCD computer screen (60 Hz refresh rate and 1680 × 1050 pixels screen resolution) using E-prime v2.0 software, with a background luminance of 20 cd/m^2^ in a free-viewing condition. The stimuli all had an identical ellipse shape with the maximum height × width of 140 mm × 180 mm which gave a visual angle of 9.93 × 12.68° when the viewing distance was fixed at 80 cm.

**Fig. 1:**
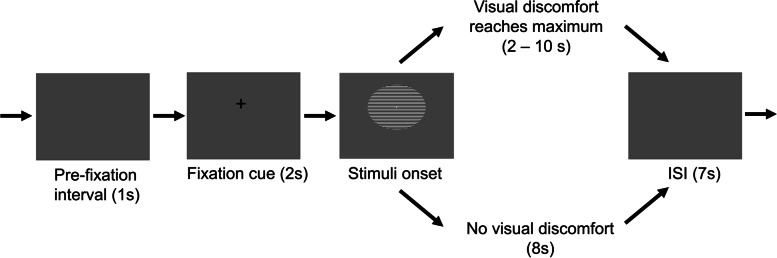
Trial sequence

Every trial started with a 1-s pre-fixation period followed by the presentation of a 2-s fixation cue at the centre of the screen prior to stimuli onset. Participants were instructed to maintain their focus at the centre of the stimuli after one of the three gratings was presented. They were also asked to either left-click with their index finger when their visual discomfort had reached the maximum (typically 2 to 10 s) or right-click with their middle finger if they did not have any forms of visual discomfort after an 8-s counting in their minds. There was a 7-s inter-stimulus interval followed by the participant’s response before the onset of the fixation of the next trial (see Fig. 1 for the trial sequence). Each type of stimuli was presented for 50 repetitions in a pseudo-random order. A total of 150 trials were separated into 10 blocks with breaks in between. To examine the effect of repeated stimulation, trial 1- 75 and trial 76 - 150 were further coded as a 2-level independent variable: 1st half and 2nd half, and later be compared in our EEG analyses.

### EEG recording and preprocessing

The 64-channel EEG signal was recorded at 500 Hz by an EEGO Sports amplifier (ANT Neuro) and Waveguard caps containing Ag/AgCl electrodes in which impedances were kept below 20 kΩ. AFz was used as ground while CPz was used as an on-line reference which was subsequently re-referenced off-line to average reference. Two pairs of bipolar EOG electrodes were used to capture the horizontal (located at the outer canthi of left and right eyes) and vertical (located at the left lid-cheek junction and above the left eyebrow) eye movements.

The preprocessing of the data was performed in Matlab using EEGLAB (version 14.1.2b) [42]. First, the raw data was bandpassed at 0.8 to 30 Hz. The EEG epochs were then locked to the onset (− 2 to 3 s) of the visual stimuli. Next, the ocular artefacts (e.g. eye blinks and eye movements) were removed using independent component analysis (ICA). After ICA pruning the data was once again inspected manually and trials with excessive noise were rejected. Finally, trials with responses given in less than 1000 ms were also removed to provide a motor-response free window for post-stimulus analyses.

### Oscillatory analysis

The data were analysed using the Fieldtrip toolbox [43]. The event-related activities were first computed by calculating the Time-frequency representations (TFRs) of power for each EEG epoch using sliding Hanning tapers with a 3-cycle time window for each frequency (ΔT = 3/f). The power spectra of the epochs were further divided into the pre-fixation cue interval (− 3 to − 2 s prior to onset of visual gratings), pre-stimulus (− 2 to 0 s prior to onset of visual gratings) and post-stimulus (0 to 1 s) intervals. The TFRs of power for the pre-fixation cue and pre-stimulus period were represented in absolute power (μV^2^) with no baseline being selected while the post-stimulus oscillatory activity was assessed in terms of change in power relative to the mean power in the baseline period − 700 to − 200 ms before the onset of the visual stimuli [44, 45].

Non-parametric cluster-based permutation analysis [46] was conducted on the pre-stimulus and post-stimulus intervals separately. In this method, the neighbouring spatiotemporal sample data were clustered if the mean amplitude differences between migraine and control exceeded the threshold of 5% significance level. The electrode-time clusters with a Monte Carlo *p*-value less than .025 (two-tailed) was considered significant (simulated by 5000 iterations), suggesting a between-group statistical difference. It is worth noting that the cluster-permutation analysis is a mass-univariate approach in which a large number of univariate tests, are used to compare the time-course of the power of alpha activity across all the scalp locations while controlling for multiple comparisons [46]. This means that our analysis was not restricted by prior scalp locations of interest.

In addition to the direct between-group comparison, we were also interested in examining the interaction effect of prolonged aversive visual stimulation and migraine condition on oscillatory activities. Therefore, the TFRs of power for pre-stimulus and post-stimulus were split into first half and second half of the experiment for further comparisons.

## Results

### Behavioural data

#### Migraine patients exhibited greater discomfort to the visual stimuli

**Fig. 2:**
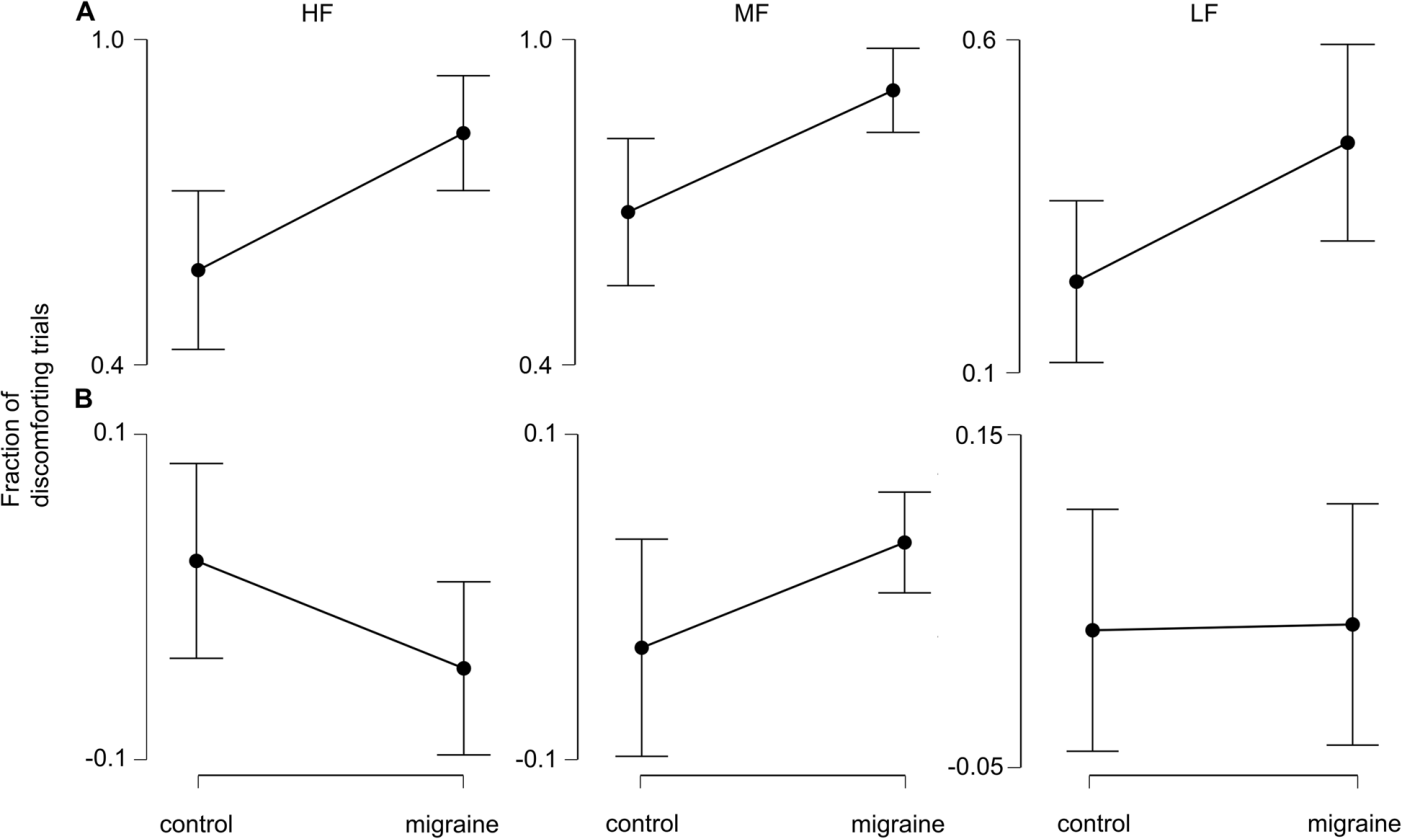
Fraction of discomforting trials for migraineurs and controls. **A** The mean fraction (with 95% CI) of discomforting trials for migraine vs. control across 3 conditions. **B** The mean change (2nd half – 1st half) of fraction (with 95% CI) of discomforting trials for migraine vs. control across 3 conditions

For each participant, the number of trials indicating discomfort (i.e left mouse clicked trials) were divided by the total number of trials, which produced the “fraction of discomforting trials” as the dependent measure for each grating condition. A two-way mixed ANOVA with repeated measures for grating condition (migraine vs. control x HF vs. MF vs. LF) was conducted. Results revealed significant main effects of migraine, *F* (1, 53) = 11.6, *p* = .001 and grating, *F* (2,106) = 48.1, *p* < .001, and no significant interaction effect, *F* < 0.2. Due to the unequal group variance and non-sphericity of the data, a non-parametric Friedman’s test was also conducted which gave a consistent result with the above parametric analysis. As post-hoc measures, Welch’s t-tests showed that migraineurs had experienced visual discomfort in more trials compared to controls in all three conditions (Fig. 2A), HF: t = 2.87, *p* = .006, (mean: 82.7% vs. 57.5%); MF: t = 2.95, *p* = .005, (mean: 93.5% vs. 71.0%); LF: t = 2.24, *p* = .029 (mean: 44.5% vs. 23.7%) (all *p*-values remained significant after false-discovery rate correction).

To investigate the effect of repeated visual stimulation, the dependent measure “change of fraction of discomforting trials” was calculated by subtracting the “fraction of discomforting trials” of the 1st half trials from the 2nd half trials. Another 2-way mixed ANOVA (migraine vs. control x HF vs. MF vs. LF) was then conducted based on this dependent measure. Despite showing no significant main effect of migraine (*F* < 1), there was a marginally significant interaction effect, *F* (2, 106) = 3.32, *p* = .04. Post-hoc tests revealed that, while not reaching significance, migraineurs did have a trend of experiencing more visual discomfort/distortions in the 2nd half of the trials for the MF condition but not HF and LF, Welch’s t = 2.00, *p* = .051, (Fig. 2B).

We also extracted the discomforting trials for all participants and conducted a repeated measure ANOVA (migraine (yes or no) x grating type (LF, MF & HF) using reaction time as dependent variable. The results showed no significant main effect nor any interaction effect (all *p* > .300).

### EEG data

#### Migraine patients had reduced alpha power relative to controls prior to onset of the visual grating

Although the main focus of the present study was the induced power changes in the alpha band (8–12 Hz), the oscillatory activities in theta (4–7 Hz) and beta (15–20 Hz) band were also examined. The frequency ranges of these bands were chosen and motivated according to prior studies [34, 47–50].

**Fig. 3:**
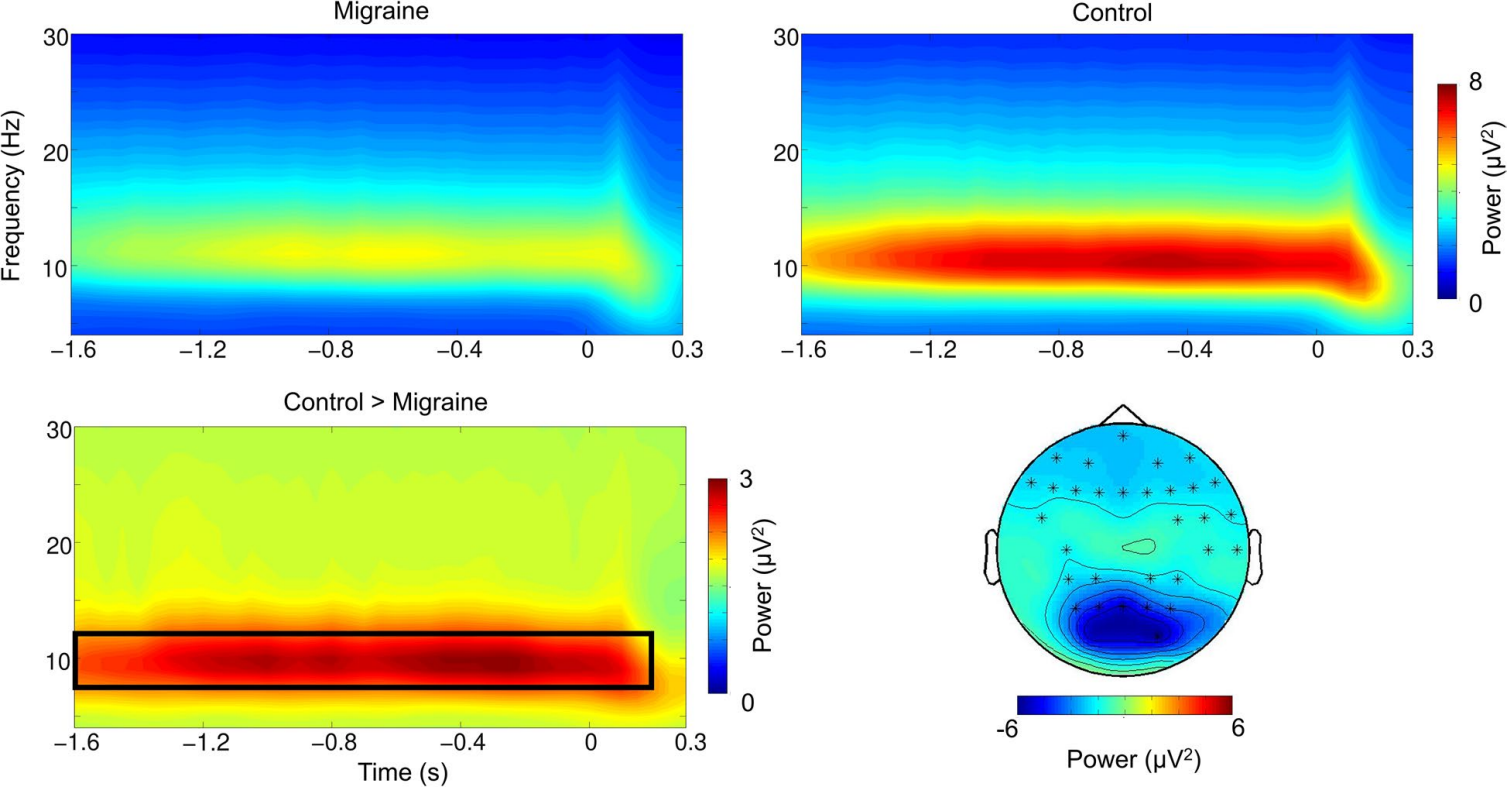
Grand mean (collapsed across all electrodes) time-frequency representation of power and topography of the alpha-band power differences (migraine - control) for the highlighted interval. The electrodes with the maximum effect over the period [−1.6 to 0.2] were highlighted with an asterisk (*)

**Fig. 4:**
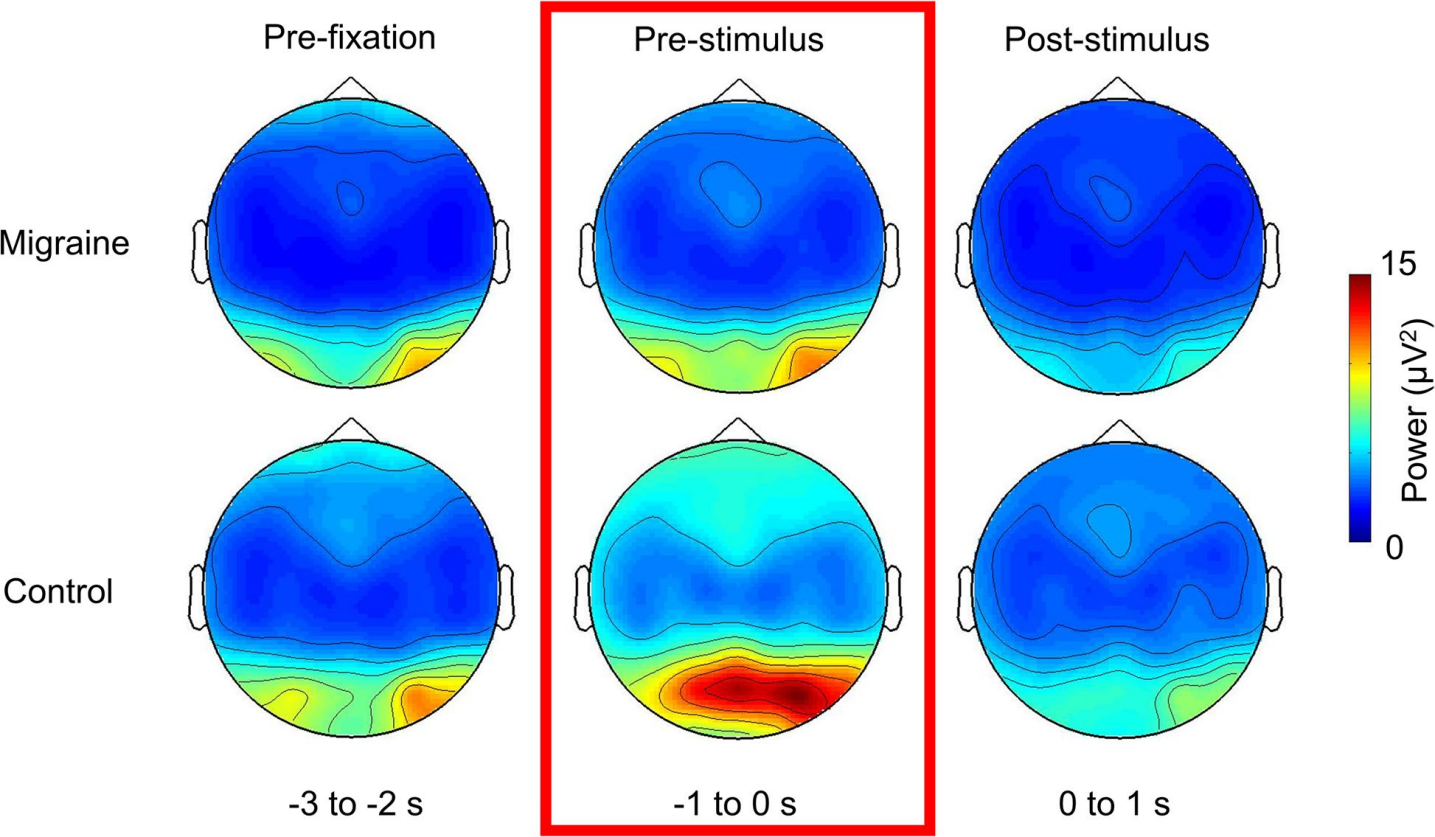
Topographies of the alpha power for pre-fixation, pre-stimulus and post-stimulus period (collapsed 3 grating conditions). The pre-stimulus alpha power for migraineurs was significantly lower than controls in the occipital-parietal region, suggesting a more excitable visual-associated cortex

Our nonparametric cluster-based permutation tests did not show any significant differences in theta, alpha and beta power between migraineurs and controls in the pre-fixation cue interval. We did however find that post-fixation cue, in the − 1.6 to 0.2 s interval relative to the onset of the visual gratings, alpha activity was significantly lower in the migraine patients relative to controls (*p* = .013; Fig. 3). The effect was most pronounced over the occipital-parietal area. While the migraine sufferers and controls did not markedly differ in their baseline level (i.e pre-fixation) of alpha activity, the significantly reduced alpha power prior to the onset of the visual grating suggested that their visual cortex was in a more excited state prior to the onset of the visual gratings (Fig. 4).

#### Both the migraineurs and controls have greater pre-stimulus alpha power in the 2nd half of the experiment

Next, in order to examine the effect of prolonged visual stimulation, we compared the pre-fixation cue alpha power and pre-stimulus alpha power in the first 75 trials of the experiment relative to trials 76 to 150.

**Fig. 5:**
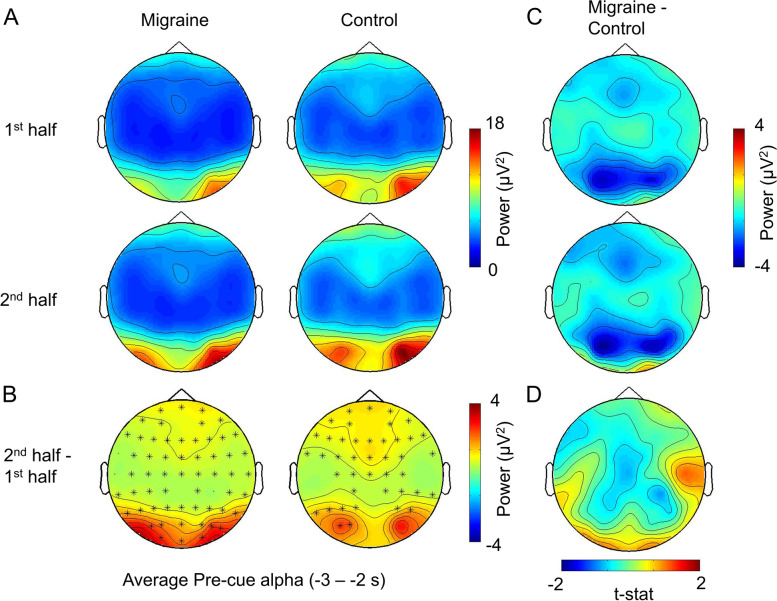
The average pre-fixation alpha power change between 1st half and 2nd half of the experiment. **A** The voltage map showed that the pre-cue alpha was the strongest in the occipital area. **B** Cluster-based permutation analysis on the pre-fixation interval (− 3 to 2 s relative to the onset of stimuli) revealed an enhanced alpha power in 2nd half of the experiment for both migraine and control. The power differences were maximal in the occipital regions (significant channels are highlighted with an asterisk (*) (migraine: *p* < .001; control: *p* = .004). **C** There was no significant difference in pre-fixation alpha between migraine and control for both 1st half and 2nd half of the experiment. **D** The alpha power increase in the 2nd half was also not significantly different between migraine and control

**Fig. 6:**
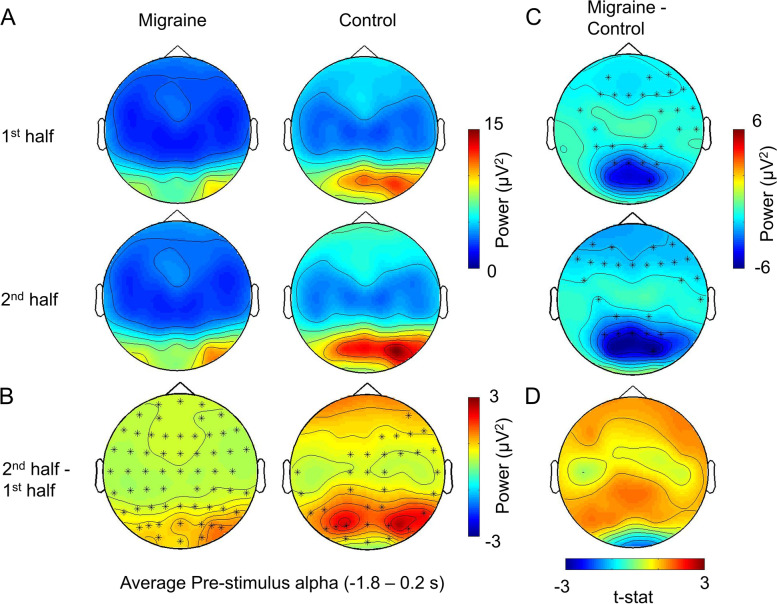
The average pre-stimulus alpha power change between 1st half and 2nd half of the experiment. **A** The voltage map showed that the pre-fixation alpha was the strongest at the occipital area. **B** Cluster-based permutation analyses displayed one significant cluster for migraine (*p* = .0002, t = − 1.8 to 0.25) and two for control (*p* = .0008, t = − 1.8 to − 0.75; *p* = .002, t = − 0.7 to 0.2). The significant channels are highlighted by an asterisk (*). **C** For the between group differences, there were one significant cluster for 1st half (*p* = .013) and one for 2nd half (*p* = .019) at the [− 1.8 to 0.2 s] interval, with the alpha power differences mainly distributed over the parietal-occipital region. **D** The pre-stimulus alpha power increase in the 2nd half was not significantly different between migraine and control groups

We found that both of the migraine group and control group had a significant increase in pre-fixation (migraine: *p* < .001; control: *p* = .004; Fig. 5) and pre-stimulus alpha-band power in the 2nd half of the experiment (migraine: *p* < .001; control: *p* < .001; Fig. 6). However, the magnitude of increase was not significantly different between migraine and control groups for both pre-fixation and pre-stimulus intervals (Figs. 5D and 6D). We observed that the pre-stimulus alpha power was consistently lower in migraineurs (Fig. 6C) for both 1st and 2nd half of the experiment (1st half, *p* = .013, 2nd half *p* = .019).

#### No difference in post-stimulus alpha suppression between migraineurs and controls

**Fig. 7:**
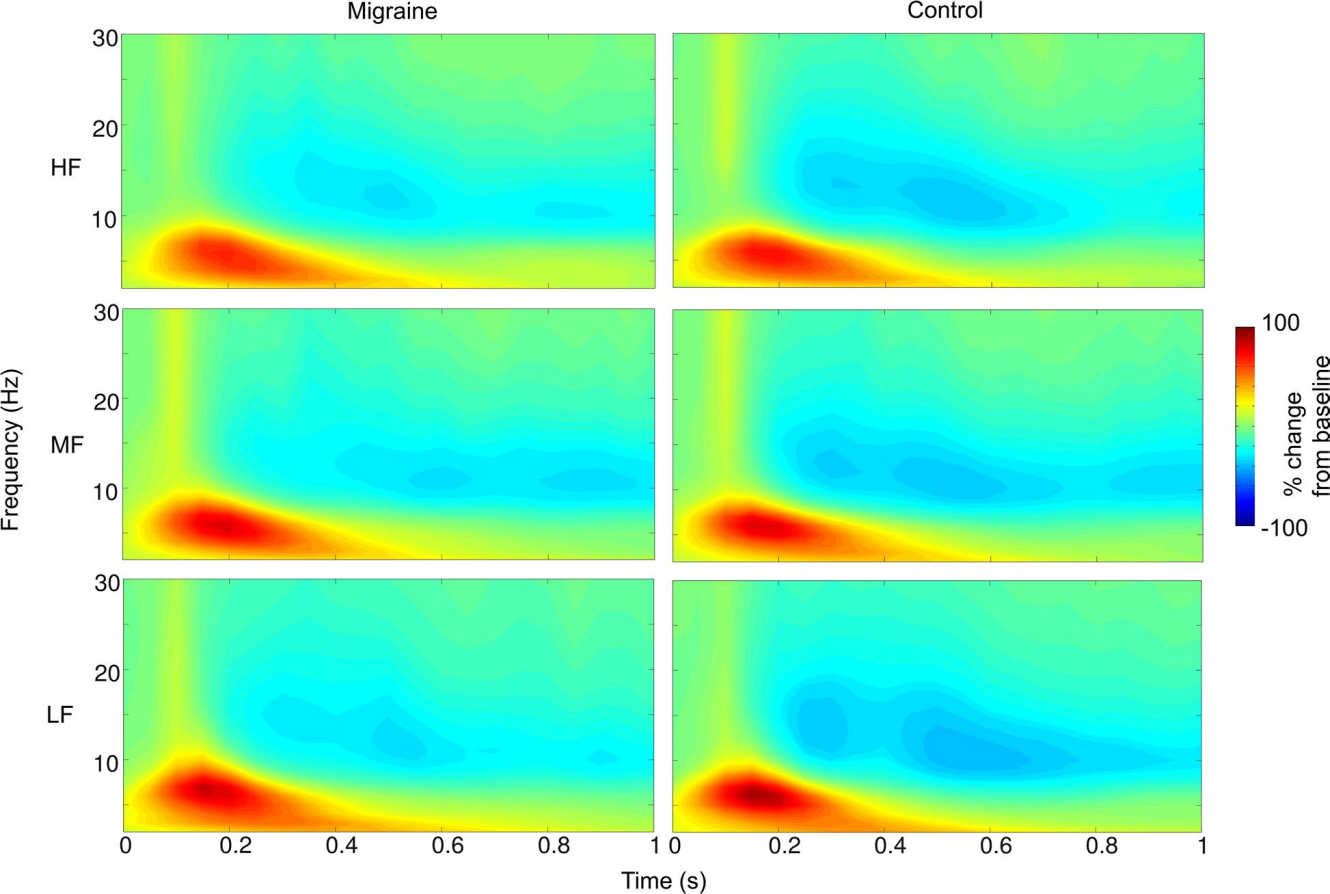
The post-stimulus percentage change of power (migraine vs. control) across the three experiment conditions (HF, MF and LF). The spectrogram indicated the percentage change of power using the pre-stimulus interval − 700 to − 200 ms before the stimulus onset as the baseline

The visual stimuli induced a theta power (4-7 Hz) increase peaking at around 200 ms after the stimulus onset in both the migraine and control groups across all three experimental conditions (HF, MF and LF; Fig. 7). There were also alpha and beta power decreases starting at 300 ms after the grating onset in all conditions. The cluster-based permutation analyses at the post-stimulus interval (0 – 1 s) did not find any significant differences between migraine and control in terms of the magnitude of alpha and beta power decrease and theta power increase across all conditions, all monte-carlo *ps* > 0.05.

#### Prolonged visual stimulation enhanced post-stimulus alpha suppression to MF gratings in migraineurs

Finally, we examined the effect of prolonged visual stimulation on post-stimulus alpha modulation. We first focused our analysis on the alpha suppression to the MF grating given that it was the grating reported to be causing the most visual discomfort.

**Fig. 8:**
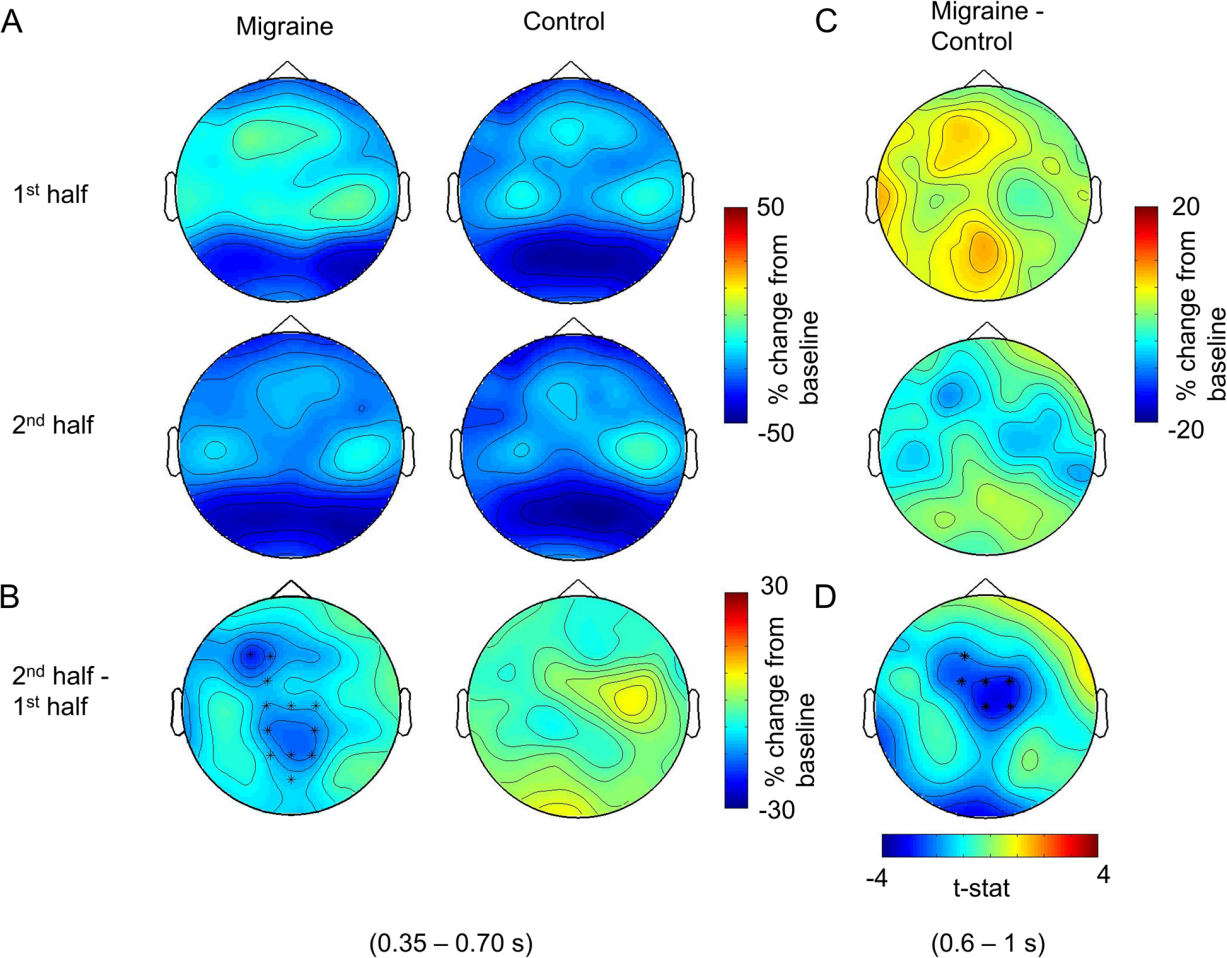
The average post-stimulus alpha power change between 1st half and 2nd half of the experiment for MF condition. **A** Cluster-based permutation analysis on the post-stimulus interval (0 to 1 s after the MF grating onset) revealed an enhanced alpha suppression in 2nd half of the experiment for migraine between 350 and 700 ms after the stimulus onset. **B** The significant channels (highlighted with *) were distributed over the central-parietal regions. **C** There was no significant difference in alpha suppression between migraine and control for both 1st half and 2nd half of the experiment. **D** The average alpha suppression (600 – 1000 ms after the stimulus onset) for migraine was stronger in the 2nd half in the central-frontal region. The significant cluster (highlighted with an asterisk (*)) indicated the maximum differences in alpha suppression between migraine and control

We found that for the migraine patients, alpha suppression to the MF grating was significantly greater in 2nd half of the experiment, 350 ms to 700 ms (*p* = .024) after the MF grating onset (Fig. 8A & B). This enhanced suppression was maximal over the central-parietal electrodes. On the other hand, the post-stimulus alpha suppression to MF grating for controls was not significantly different between the 1st half and 2nd half of the experiment.

To further evaluate the interaction effect of migraine and repeated stimulation on alpha suppression, the alpha power change of 2nd half was subtracted from the 1st half separately for migraine and the control. The resultant data were then subjected to another cluster-based permutation analysis to obtain the between-group effect (migraine vs. control). The result revealed a marginally significant cluster with the effect maximally distributed over central areas, *p* = .036 around 0.6 to 1 s after the MF grating onset, indicating a stronger alpha suppression in the 2nd half of the experiment for migraine (Fig. 8D).

We repeated the above analyses for the HF and LF gratings, however, we did not observe a significant difference in alpha suppression between 2nd and 1st half of the experiment neither in the migraineurs nor controls. We also conducted the same analyses for theta and beta activity, which also did not yield any significant differences between migraineurs and controls.

## Discussion

In the present study, we used the modulation of ongoing alpha activity induced by the onset of visual stimuli to assess the excitability of the visual cortex of migraine patients in anticipation of a visual grating, as well as during its processing. We also examined how the alpha modulation during these periods changed from the 1st to 2nd half of the experiment, which allowed us to assess the impact of prolonged visual stimulation. We focused our investigation on modulations of oscillatory activity in the alpha range given that previous work had found support of this rhythm to be involved in the gating of visual input [34]. There were no significant differences in baseline alpha power between migraineurs and non-migraine controls. In contrast, alpha power was reliably reduced in migraineurs during the pre-stimulus period prior to the expected onset of the visual gratings. We did not observe an overall difference in the post-stimulus suppression of alpha activity between migraineurs and non-migraineurs. However, we did observe that migraineurs had significantly more alpha suppression to the visual grating associated with the greatest visual discomfort (MF grating), in the 2nd half of the experiment. We interpret the lower pre-stimulus alpha power seen in migraineurs as reflecting that their visual cortex is in a more excitable state in anticipation of the arrival of the visual stimuli perhaps via a disrupted perceptual learning mechanism. Moreover, the increased alpha suppression to the MF observed in this group suggests that their visual cortex is hyperresponsive to repeated stimulation. We will now discuss these findings in greater detail.

### Alpha power in pre-stimulus period

Migraineurs persistently showed a pre-stimulus alpha power deficit maximally covering the occipital regions of the brain. During the pre-stimulus period, participants were required to maintain their vision on a steady fixation point, which functioned as a visual cue to hint the onset of the visual stimuli. With the current setup, the visual target would always appear at the same temporal and spatial position which made the stimulus onset being completely predictable. Such dominance of alpha-band oscillations in the pre-stimulus interval was expected and also in-line with previous literature in which alpha rhythm was found to predict visual detection [51], discrimination [35], awareness [52] and the induction of phosphenes [53]. In these visual experiments, researchers found that the phase angle and power of alpha oscillations preceding the missed or detected visual targets were significantly different [51, 54]. As a result, some researchers proposed that the sensory system was modulated to an ideal “excitability state” by top-down temporal prediction and therefore, alpha-band oscillations might indicate the “excitability state” of the sensory system. Functionally speaking, alpha oscillations might activate the local inhibitory neurons at the visual cortex in order to suppress/filter excessive visual input [55–57]. Therefore, with diminished alpha-band activities, more sensory neurons might be activated. Additionally, pre-stimulus occipital alpha was found to indicate the enhanced excitability of the visual cortex [58].

Interestingly, in more demanding visual detection tasks, the detection of a target was associated with a decrease in pre-stimulus alpha-band power [51, 59, 60]. In our study, we used a non-cognitive demanding task together with aversive stimuli, where pre-stimulus alpha-power was instead tuned to a higher level. We speculated that such an increment of alpha power could re-adjust the sensory cortex into a suitable excitability state after the higher cortical area eventually learnt that the stimuli were irritating. We suggested that such a top-down guided perceptual learning process [61] was beneficial to the participant since deactivating the sensory system might relieve the discomforting sensation brought by the visual stimulation from the gratings.

Based on the behavioural data, migraineurs manifested more visual discomfort in response to all types of gratings. An intact alpha modulated neuronal circuit should exhibit an inhibited excitability state. Therefore, it was a piece of clear evidence showing that migraineurs had an impairment with a lower ceiling of alpha-band power despite knowing that the upcoming visual stimulation would be aversive. With alpha power known to be associated with the peak amplitude of event-related potential components, for example, P1 [62], N1-P2 [63] and P3 [59], the reduced pre-stimulus alpha on migraineurs in this experiment appeared to be consistent with the abnormal increase of VEP components found in the early and recent literatures [3, 23, 64, 65]. Additionally, as we suggested, a lower peak alpha might influence the visual perceptual learning process, which could be associated with the poorer performance of migraine patients in certain visual tasks where they must learn to suppress the visual noise in order to perform [66, 67]. Another symptom of migraine - photosensitivity was also linked to decreased posterior alpha activities [68]. Collectively, our studies together with the findings in previous literatures all support the visual hyperresponsiveness of migraineurs, which could be driven by the dysfunction of alpha-band activity regulation.

### The inhibitory account of alpha activity

While there is currently an abundance of empirical support for alpha inhibition, it is still currently unknown exactly how this inhibition occurs at the neuronal level. There is evidence that alpha could be inhibiting the firing rate of neurons [69]. Moreover, the phase of the ongoing alpha activity appears to be linked with the amplitude of high-frequency activity in the gamma range (30 – 200 Hz) across laminar layers in primary visual cortex (V1), suggesting that the alpha cycle could function as ‘gain-control’ through limiting the duty-cycle of visual processing [70].

Currently, it is widely believed that thalamus may play a critical role in generating alpha rhythms (for review see [71]). Specifically, the interaction between the lateral thalamic nuclei and the nucleus reticularis of the thalamus has been proposed to serve as a key ‘hub’ in pacing the speed of cortical alpha activity [72, 73]. There have been suggestions that alpha might serve as a feedback signal from the cortex that could modulate the neural excitability in the thalamo-recipient layer [34]. While the fluctuation in alpha power could be driven by top-down task demands, they can also occur due to multiple factors such as changes in arousal [74].

### Hyperresponsive specifically to grating in medium frequency

In the current experiment, participants had to make a behavioural response in every trial in respect of their visual experiences. Though main effects of migraine on the extent of alpha suppression was lacking, both groups demonstrated stronger cortical activations probably due to an increase in visual gain or spatial attention starting from 400 ms after the stimulus onset [75, 76]. Such reduction of alpha might also be associated with a local change of cerebral metabolic rate [77]. As the experiment progressed, there was a general increase of baseline alpha power for both migraine and control. Without any significant group differences, such an effect might not be associated with migraine pathology (and is perhaps due to fatigue) [78], therefore, it was not the main focus of the present study.

Apart from this, we observed that the alpha suppression/sensitisation topography was maximal over the occipital electrodes(see Fig. 8A). More interestingly, migraine patients displayed an enhanced alpha suppression specifically to grating in medium frequency (3 cpd) after repeated stimulations. These findings are new and have not been reported in the literature previously. Here we make the following tentative interpretations. First, this effect could be indirectly caused by cognitive fatigue rather than sensitisation. Mathematically speaking, when there was an increase in baseline and pre-stimulus alpha, a similar/unchanged level of post-stimulus alpha and cortical activations at the 2nd half of the experiment would appear as a stronger alpha suppression relatively. However, we did not observe the same effect over the control group and the other experimental conditions (HF & LF), thus, it is not likely that the present findings were mainly driven by a kind of knock-on effect. Another possibility was that the “stronger” alpha suppression of 2nd half trials was indeed a disguise of the “weaker” alpha suppression in the 1st half. In other words, such a phenomenon indicated a recovery of alpha suppression of the migraine sufferers. However, if the alpha suppression in the 2nd half represented a “back to normal” excitability state for migraineurs, we would expect a decrease in visual discomfort rather than the observed increase. Therefore, we believe that the diminished alpha suppression in the 1st half was the “better” state for migraineurs in which the excitability was selectively suppressed in order to reduce the aversive effect of the MF grating. Such sensory process could be a similar perceptual learning mechanism to that we discussed earlier, which was disrupted in the 2nd half of the experiment due to repeated visual stimulations leading to the elevation of alpha suppression. Since migraineurs also reported experiencing visual discomfort in more trials during the 2nd half, this hypothesis accords better to both the behavioural and electrophysiological data.

Additionally, it is consistent with the “habituation deficit” phenomenon found in migraine. Habituation deficit highlighted the improper perceptual learning process accompanied by neuroplasticity, where repeated visual stimulations do not produce a suppressed visual responses [79, 80]. This characteristic, which is contrary to the finding in the healthy population, can be commonly seen in migraineurs and often reflected by an unchanged/enhanced rather than a reduced VEP [14, 22, 81]. Habituation, which was proposed as an adaptive cortical mechanism mediated by GABAergic inhibitory interneurons [82, 83], occurs in order to prevent the sensory cortex from overstimulation and lactate accumulation [17]. It is possible that gratings (especially in medium frequency) might stimulate a relatively localised nerve network of the primary visual cortex (see a review of pattern glare [33]), thus, the long-term exposure to the MF grating might overload the synthesis or reuptake of the inhibitory neurotransmitter of the impaired GABAergic system of migraineurs.

### Limitation and future direction

Previous literature has suggested that GABAergic feedback from interneurons plays a critical role in the physiological mechanism generating the alpha rhythm [84–86]. Although speculative, the rhythmic activity generating the alpha rhythm could be because of GABAergic inhibitory feedback paced by neocortical or thalamic rhythm generators [71, 87, 88]. This could underlie the inhibitory nature of alpha activity, where GABAergic feedback reduces excitatory input, or silences processing in pyramidal neurons [34].

The impairment of GABAergic mediated inhibitory network on migraine has been widely discussed in previous literatures [89, 90]. Apart from visual disturbances, a dysfunctional GABAergic system, is more susceptible to enhanced synaptic transmission, spreading depression [80] and the activations of trigeminal nociceptive neurons are all possible causes of the head pain of migraine attack [91, 92]. Although migraine research in GABAergic pathway facilitates prophylactic medication development targeting GABA receptors [93, 94], some research showed that the concentration of GABA between migraineurs and healthy controls were not fundamentally different [95]. In addition, serotonin appeared to be associated with the above network since the treatment of anti-reuptake agents of serotonin were able to restore the function of habituation [96]. Being the most abundant interneurons of the cerebral cortex, GABAergic interneurons are also known to associate with most cognitive functions that are not unique to the pathology of migraine. Therefore, a deeper investigation at functional, anatomical and even the cellular level of GABAergic interneuron might be critical to understand the cause of migraine in the future.

It should be noted that visual disturbances, hyperreponsiveness and alpha-power deficit by themselves likely cannot provide a full picture of migraine pathophysiology. Moreover, the origin of the alpha-power deficit and how it is associated with the neuropathology of migraine is unknown. Nonetheless, the maximum effect of alpha power differences we found was around the parietal areas rather than localising at the occipital regions which directly received sensory input from the early visual pathways. It is rather not surprising since recent studies havealready shown that the occipital alpha and the neural activity of the visual cortex could be modulated by both cortico-cortical (e.g. prefrontal, parietal) and thalamocortical interactions [88, 97]. Recent development on predictive coding and perceptual learning also challenged the idea of perception being a pure bottom-up process, but a bi-directional and hierarchical integration of information from both the higher-order cortical area and lower-order subcortical area [98, 99]. In this sense, an abnormality of visual sensations such as visual disturbances does not necessarily suggest overt damage to the visual cortex, any inter-connected network could contribute to such sensory impairments.

In the current experiment, participants’ eye movements were not monitored using an eye-tracker, which meant we were not able unequivocally to rule out that the participants were fixated on the stimuli in every trial. While we did use EOG electrodes to reject trials with an overt eye movement, future research employing an eye-tracker would afford the possibility to reject trials where the participants’ eyes were not fixed on the stimuli.

In conclusion, our study revealed that migraine patients had pre-stimulus alpha deficit during the anticipation of the visual stimulation. They also manifested increased alpha suppression selectively to the grating with a spatial frequency of 3 cpd after repeated stimulation. Given that alpha activity has been associated with the functional inhibition of sensory cortices, the present study is consistant with the view that migraine patients have a hyperresponsive visual cortex. We speculated that this hyperresponsivenesss could be the consequence of an improper perceptual learning process driven by the dysfunction of GABAergic inhibitory mechanism. Taken together, our study showed converging behavioural and electrophysiological evidence for the hyperresponsiveness of migraine sufferers which could underlie their experience of visual disturbances.

## Supplementary Information

**Additional file 1: Table S1.** The clinical characteristics of the 28 migraine patients included in the data analyses. **Table S2.** Sample characteristic of the 29 migraine-free controls.

## Data Availability

All the data and analysis scripts will be made available after acceptance.

## Abbreviations

TMS: Transcranial magnetic stimulation
cpd: Cycles per degree
VEPs: Visual evoked potentials

## References

[1] Boulloche N, Denuelle M, Payoux P (2010). Photophobia in migraine: an interictal PET study of cortical hyperexcitability and its modulation by pain. J Neurol Neurosurg Psychiatry.

[2] Palmer JE, Chronicle EP, Rolan P, Mulleners WM (2000). Cortical hyperexcitability is cortical under-inhibition: evidence from a novel functional test of migraine patients. Cephalalgia.

[3] Fong CY, Law WHC, Braithwaite J, Mazaheri A (2020). Differences in early and late pattern-onset visual-evoked potentials between self- reported migraineurs and controls. NeuroImage Clin.

[4] Haigh SM, Karanovic O, Wilkinson F, Wilkins AJ (2012). Cortical hyperexcitability in migraine and aversion to patterns. Cephalalgia.

[5] van der Kamp W, Maassen VanDenBrink A, Ferrari MD, van Dijk JG (1996). Interictal cortical hyperexcitability in migraine patients demonstrated with transcranial magnetic stimulation. J Neurol Sci.

[6] Aurora SK, Cao Y, Bowyer SM, Welch KMA (1999). The occipital cortex is Hyperexcitable in migraine: experimental evidence. Headache: the journal of head and face. Pain.

[7] Aurora SK, Welch KMA, Al-Sayed F (2003). The threshold for phosphenes is lower in migraine. Cephalalgia.

[8] Fumal A, Bohotin V, Vandenheede M (2003). Effects of repetitive transcranial magnetic stimulation on visual evoked potentials: new insights in healthy subjects. Exp Brain Res.

[9] Fong CY, Takahashi C, Braithwaite JJ (2019). Evidence for distinct clusters of diverse anomalous experiences and their selective association with signs of elevated cortical hyperexcitability. Conscious Cogn.

[10] Wilkins A, Nimmo-Smith I, Tait A (1984). A neurological basis for visual discomfort. Brain.

[11] Wilkins AJ (1995). Visual stress.

[12] McKendrick AM, Chan YM, Vingrys AJ (2018). Daily vision testing can expose the prodromal phase of migraine. Cephalalgia.

[13] Mulleners WM, Chronicle EP, Palmer JE (2001). Visual cortex excitability in migraine with and without Aura. Headache: the journal of head and face. Pain.

[14] Schoenen J, Wang W, Albert A, Delwaide PJ (1995). Potentiation instead of habituation characterizes visual evoked potentials in migraine patients between attacks. Eur J Neurol.

[15] Ambrosini A, Coppola G, Iezzi E (2016). Reliability and repeatability of testing visual evoked potential habituation in migraine: a blinded case–control study. Cephalalgia.

[16] Coppola G, Pierelli F, Schoenen J (2009). Habituation and migraine. Neurobiol Learn Mem.

[17] Sappey-Marinier D, Calabrese G, Fein G (1992). Effect of photic stimulation on human visual cortex lactate and phosphates using1H and31P magnetic resonance spectroscopy. J Cereb Blood Flow Metab.

[18] Thompson RF (2009). Habituation: a history. Neurobiol Learn Mem.

[19] Restuccia D, Vollono C, Virdis D (2014). Patterns of habituation and clinical fluctuations in migraine. Cephalalgia.

[20] Ambrosini A, de Noordhout AM, Sándor PS, Schoenen J (2003). Electrophysiological studies in migraine: a comprehensive review of their interest and limitations. Cephalalgia.

[21] Ambrosini A, Schoenen J (2003). The electrophysiology of migraine. Curr Opin Neurol.

[22] Áfra J, Cecchini AP, de Pasqua V (1998). Visual evoked potentials during long periods of pattern-reversal stimulation in migraine. Brain.

[23] Oelkers R, Grosser K, Lang E (1999). Visual evoked potentials in migraine patients: alterations depend on pattern spatial frequency. Brain.

[24] Shibata K, Osawa M, Iwata M (1997). Pattern reversal visual evoked potentials in classic and common migraine. J Neurol Sci.

[25] Shibata K, Osawa M, Iwata M (1998). Pattern reversal visual evoked potentials in migraine with aura and migraine aura without headache. Cephalalgia.

[26] Haigh SM, Chamanzar A, Grover P, Behrmann M (2019). Cortical hyper-excitability in migraine in response to chromatic patterns. Headache: the journal of head and face. Pain.

[27] Lia C, Carenini L, Degioz C, Bottachi E (1995). Computerized EEG analysis in migraine patients. Ital J Neurol Sci.

[28] Bjørk MH, Stovner LJ, Engstrøm M (2009). Interictal quantitative EEG in migraine: a blinded controlled study. J Headache Pain.

[29] O’Hare L, Menchinelli F, Durrant SJ (2018). Resting-state alpha-band oscillations in migraine. Perception.

[30] Cao Z, Lin CT, Chuang CH (2016). Resting-state EEG power and coherence vary between migraine phases. J Headache Pain.

[31] Chamanzar A, Haigh SM, Grover P, Behrmann M (2021). Abnormalities in cortical pattern of coherence in migraine detected using ultra high-density EEG. Brain. Communications.

[32] Sand T, Zhitniy N, White LR, Stovner LJ (2008). Brainstem auditory-evoked potential habituation and intensity-dependence related to serotonin metabolism in migraine: a longitudinal study. Clin Neurophysiol.

[33] Evans BJW, Stevenson SJ (2008). The pattern glare test: a review and determination of normative values. Ophthalmic Physiol Opt.

[34] Jensen O, Mazaheri A (2010) Shaping functional architecture by oscillatory alpha activity: gating by inhibition. Front Hum Neurosci. 10.3389/fnhum.2010.00186 10.3389/fnhum.2010.00186 PMC2990626 21119777

[35] van Dijk H, Schoffelen JM, Oostenveld R, Jensen O (2008). Prestimulus oscillatory activity in the alpha band predicts visual discrimination ability. J Neurosci.

[36] van Diepen RM, Foxe JJ, Mazaheri A (2019). The functional role of alpha-band activity in attentional processing: the current zeitgeist and future outlook. Curr Opin Psychol.

[37] Foxe JJ, Snyder AC (2011). The role of alpha-band brain oscillations as a sensory suppression mechanism during selective attention. Front Psychol.

[38] Klimesch W, Sauseng P, Hanslmayr S (2007). EEG alpha oscillations: the inhibition-timing hypothesis. Brain Res Rev.

[39] Haigh SM, Cooper NR, Wilkins AJ (2018). Chromaticity separation and the alpha response. Neuropsychologia.

[40] Babiloni C, Brancucci A, Percio C del, et al (2006) Anticipatory electroencephalography alpha rhythm predicts subjective perception of pain intensity. J Pain 7:709–717. 10.1016/j.jpain.2006.03.005 10.1016/j.jpain.2006.03.005 17018331

[41] Olesen J (2018). International classification of headache disorders. Lancet Neurol.

[42] Delorme A, Makeig S (2004). EEGLAB: an open source toolbox for analysis of single-trial EEG dynamics including independent component analysis. J Neurosci Methods.

[43] Oostenveld R, Fries P, Maris E, Schoffelen JM (2011) FieldTrip: open source software for advanced analysis of MEG, EEG, and invasive electrophysiological data. Comput Intell Neurosci 2011. 10.1155/2011/156869 10.1155/2011/156869 PMC3021840 21253357

[44] van Diepen RM, Cohen MX, Denys D, Mazaheri A (2015). Attention and temporal expectations modulate power, not phase, of ongoing alpha oscillations. J Cogn Neurosci.

[45] Mazaheri A, Segaert K, Olichney J (2018). EEG oscillations during word processing predict MCI conversion to Alzheimer’s disease. NeuroImage Clin.

[46] Maris E, Oostenveld R (2007). Nonparametric statistical testing of EEG- and MEG-data. J Neurosci Methods.

[47] Klimesch W, Doppelmayr M, Pachinger T, Ripper B (1997). Brain oscillations and human memory: EEG correlates in the upper alpha and theta band. Neurosci Lett.

[48] Rommers J, Dickson DS, Norton JJS (2017). Alpha and theta band dynamics related to sentential constraint and word expectancy. Lang Cogn Neurosci.

[49] Sauseng P, Klimesch W, Freunberger R (2006). Relevance of EEG alpha and theta oscillations during task switching. Exp Brain Res.

[50] Addante RJ, Watrous AJ, Yonelinas AP (2011). Prestimulus theta activity predicts correct source memory retrieval. Proc Natl Acad Sci U S A.

[51] Busch NA, Dubois J, VanRullen R (2009). The phase of ongoing EEG oscillations predicts visual perception. J Neurosci.

[52] Mathewson KE, Gratton G, Fabiani M (2009). To see or not to see: Prestimulus α phase predicts visual awareness. J Neurosci.

[53] Dugué L, Marque P, VanRullen R (2011). The phase of ongoing oscillations mediates the causal relation between brain excitation and visual perception. J Neurosci.

[54] Samaha J, Bauer P, Cimaroli S, Postle BR (2015). Top-down control of the phase of alpha-band oscillations as a mechanism for temporal prediction. Proc Natl Acad Sci U S A.

[55] van Kerkoerle T, Self MW, Dagnino B (2014). Alpha and gamma oscillations characterize feedback and feedforward processing in monkey visual cortex. Proc Natl Acad Sci U S A.

[56] Olsen SR, Bortone DS, Adesnik H, Scanziani M (2012). Gain control by layer six in cortical circuits of vision. Nature.

[57] Clayton MS, Yeung N, Cohen Kadosh R (2018). The many characters of visual alpha oscillations. Eur J Neurosci.

[58] Lange J, Oostenveld R, Fries P (2013). Reduced occipital alpha power indexes enhanced excitability rather than improved visual perception. J Neurosci.

[59] Ergenoglu T, Demiralp T, Bayraktaroglu Z (2004). Alpha rhythm of the EEG modulates visual detection performance in humans. Cogn Brain Res.

[60] Bauer M, Stenner MP, Friston KJ, Dolan RJ (2014). Attentional modulation of alpha/beta and gamma oscillations reflect functionally distinct processes. J Neurosci.

[61] Ahissar M, Hochstein S (2004). The reverse hierarchy theory of visual perceptual learning. Trends Cogn Sci.

[62] Fellinger R, Klimesch W, Gruber W (2011). Pre-stimulus alpha phase-alignment predicts P1-amplitude. Brain Res Bull.

[63] Brandt ME, Jansen BH (1991). The relationship between prestimulus alpha amplitude and visual evoked potential amplitude. Int J Neurosci.

[64] Sand T, Zhitniy N, White LR, Stovner LJ (2008). Visual evoked potential latency, amplitude and habituation in migraine: a longitudinal study. Clin Neurophysiol.

[65] Diener HC, Ndosi NK, Koletzki E, Langohr D (1985). Visual evoked potentials in migraine. Updating in headache.

[66] Wagner D, Manahilov V, Loffler G (2010). Visual noise selectively degrades vision in migraine. Investig Ophthalmol Vis Sci.

[67] Tibber MS, Kelly MG, Jansari A (2014). An inability to exclude visual noise in migraine. Investig Ophthalmol Vis Sci.

[68] Vaudano AE, Ruggieri A, Avanzini P (2017). Photosensitive epilepsy is associated with reduced inhibition of alpha rhythm generating networks. Brain.

[69] Haegens S, Nácher V, Luna R (2011). α-Oscillations in the monkey sensorimotor network influence discrimination performance by rhythmical inhibition of neuronal spiking. Proc Natl Acad Sci U S A.

[70] Spaak E, Bonnefond M, Maier A (2012). Layer-specific entrainment of γ-band neural activity by the α rhythm in monkey visual cortex. Curr Biol.

[71] Hughes SW, Crunelli V (2005). Thalamic mechanisms of EEG alpha rhythms and their pathological implications. Neuroscientist.

[72] Steriade M (2000). Corticothalamic resonance, states of vigilance and mentation. Neuroscience.

[73] Steriade M (2001). Impact of network activities on neuronal properties in Corticothalamic systems. J Neurophysiol.

[74] Cantero JL, Atienza M, Gómez C, Salas RM (1999). Spectral structure and brain mapping of human alpha activities in different arousal states. Neuropsychobiology.

[75] Rihs TA, Michel CM, Thut G (2007). Mechanisms of selective inhibition in visual spatial attention are indexed by α-band EEG synchronization. Eur J Neurosci.

[76] Peterson EJ, Voytek B (2017) Alpha oscillations control cortical gain by modulating excitatory-inhibitory background activity. bioRxiv:185074

[77] Cook IA, O’Hara R, Uijtdehaage SHJ (1998). Assessing the accuracy of topographic EEG mapping for determining local brain function. Electroencephalogr Clin Neurophysiol.

[78] Boksem MAS, Meijman TF, Lorist MM (2005). Effects of mental fatigue on attention: an ERP study. Cogn Brain Res.

[79] Sale A, de Pasquale R, Bonaccorsi J (2011). Visual perceptual learning induces long-term potentiation in the visual cortex. Neuroscience.

[80] Dilekoz E, Houben T, Eikermann-Haerter K (2015). Migraine mutations impair hippocampal learning despite enhanced long-term potentiation. J Neurosci.

[81] Ambrosini A, Rossi P, de Pasqua V (2003). Lack of habituation causes high intensity dependence of auditory evoked cortical potentials in migraine. Brain.

[82] Ramaswami M (2014). Network plasticity in adaptive filtering and behavioral habituation. Neuron.

[83] Giovannini MG, Rakovska A, Benton RS (2001). Effects of novelty and habituation on acetylcholine, GABA, and glutamate release from the frontal cortex and hippocampus of freely moving rats. Neuroscience.

[84] Lopes da Silva FH, van Rotterdam A, Barts P, Corner MA, Swaab DFBT-P in BR (1976). Models of neuronal populations: the basic mechanisms of rhythmicity. Perspectives in Brain Research.

[85] Crunelli V, Leresche N (1991). A role for GABAB receptors in excitation and inhibition of thalamocortical cells. Trends Neurosci.

[86] Jones SR, Pinto DJ, Kaper TJ, Kopell N (2000). Alpha-frequency rhythms desynchronize over long cortical distances: a modeling study. J Comput Neurosci.

[87] Lörincz ML, Crunelli V, Hughes SW (2008). Cellular dynamics of cholinergically induced alpha (8-13 Hz) rhythms in sensory thalamic nuclei in vitro. J Neurosci.

[88] Lorincz ML, Kékesi KA, Juhász G (2009). Temporal framing of thalamic relay-mode firing by phasic inhibition during the alpha rhythm. Neuron.

[89] Aurora SK, Al-Sayeed F, Welch KMA (1999). The cortical silent period is shortened in migraine with aura. Cephalalgia.

[90] Brighina F, Palermo A, Fierro B (2009). Cortical inhibition and habituation to evoked potentials: relevance for pathophysiology of migraine. J Headache Pain.

[91] Cornelison LE, Woodman SE, Durham PL (2020). Inhibition of trigeminal nociception by non-invasive Vagus nerve stimulation: investigating the role of GABAergic and serotonergic pathways in a model of episodic migraine. Front Neurol.

[92] Knight YE, Bartsch T, Kaube H, Goadsby PJ (2002) P/Q-type calcium-channel blockade in the periaqueductal gray facilitates trigeminal nociception: a functional genetic link for migraine? J Neurosci 22. 10.1523/jneurosci.22-05-j0002.2002 10.1523/JNEUROSCI.22-05-j0002.2002 PMC6758884 11880534

[93] Sprenger T, Viana M, Tassorelli C (2018). Current prophylactic medications for migraine and their potential mechanisms of action. Neurotherapeutics.

[94] Cutrer FM, Moskowitz MA (1996). The actions of valproate and Neurosteroids in a model of trigeminal pain. Headache: the journal of head and face. Pain.

[95] Chan YM, Pitchaimuthu K, Wu Q-Z (2019). Relating excitatory and inhibitory neurochemicals to visual perception: a magnetic resonance study of occipital cortex between migraine events. PLoS One.

[96] Ozkul Y, Bozlar S (2002). Effects of fluoxetine on habituation of pattern reversal visually evoked potentials in migraine prophylaxis. Headache: the journal of head and face. Pain.

[97] Helfrich RF, Huang M, Wilson G, Knight RT (2017). Prefrontal cortex modulates posterior alpha oscillations during top-down guided visual perception. Proc Natl Acad Sci U S A.

[98] Fong CY, Law WHC, Uka T, Koike S (2020). Auditory mismatch negativity under predictive coding framework and its role in psychotic disorders. Front Psychiatry.

[99] Rao RPN, Ballard DH (1999). Predictive coding in the visual cortex: a functional interpretation of some extra-classical receptive-field effects. Nat Neurosci.

