# Supplementary material for: Attenuated alpha oscillation and hyperresponsiveness reveals impaired perceptual learning in migraineurs
Source: J Headache Pain. 2022 Apr 5;23(1):44. doi: 10.1186/s10194-022-01410-2 (PMC8981672; doi:10.1186/s10194-022-01410-2)
Supplement: Supplementary file 1 — Additional file 1: Table S1. The clinical characteristics of the 28 migraine patients included in the data analyses. Table S2. Sample characteristic of the 29 migraine-free controls. [file 10194_2022_1410_MOESM1_ESM.docx]

Table S1. The clinical characteristics of the 28 migraine patients included in the data analyses

| subject | age | migraine type | attack frequency | pain intensity (0 – 1) | last migraine attack |
| --- | --- | --- | --- | --- | --- |
| 1 | 19 | with aura | Q3M | 0.6 | < 3 months |
| 2 | 20 |  | Q6M | 0.6 | < 6 months |
| 3 | 27 |  | Q1M | 0.8 | < 3 months |
| 4 | 20 |  | Q1M | 0.8 | < 1 month |
| 5 | 29 |  | Yearly | 0.7 | < 6 months |
| 6 | 30 |  | Q1M | 1 | < 1 month |
| 7 | 24 |  | Q6M | 0.2 | < 6 months |
| 8 | 18 |  | Q3M | 0.4 | < 6 months |
| 9 | 19 |  | Q1M | 0.5 | < 1 month |
| 10 | 19 |  | Q3M | 1 | < 6 months |
| 11 | 18 |  | Q6M | 0.1 | < 3 months |
| 12 | 21 |  | Q1M | 0.8 | < 3 months |
| 13 | 20 |  | Weekly | 1 | < 1 month |
| 14 | 19 |  | Q6M | 0.3 | < 1 month |
| 15 | 20 |  | Q6M | 1 | < 3 months |
| 16 | 19 |  | Yearly | 0.9 | > 1 year |
| 17 | 19 |  | Q3M | 0.8 | < 1 month |
| 18 | 18 | without aura | Yearly | 1 | > 1 year |
| 19 | 20 |  | Q1M | 0 | < 1 month |
| 20 | 32 |  | Not specific | 0.7 | < 3 months |
| 21 | 19 |  | Q1M | 0.3 | < 1 month |
| 22 | 18 |  | Q1M | 0.8 | < 1 month |
| 23 | 19 |  | Not specific | 1 | < 6 months |
| 24 | 19 |  | Q1M | 0.8 | > 1 week |
| 25 | 18 |  | Q3M | 0.2 | < 1 month |
| 26 | 21 |  | Q3M | 0.6 | < 3 months |
| 27 | 21 |  | Q3M | 0.5 | < 1 month |
| 28 | 20 |  | Q3M | 1 | < 6 months |

Note: Only included participants with no migraine attack before 1 week and after at least 2 weeks of the recordings; Q1M = Every month; Q3M = Every 3 months; Q6M = Every 6 months

Table S2. Sample characteristic of the 29 migraine-free controls.

| subject | age | Headache  Frequency (1 - 4)* | Any family member having migraine? |
| --- | --- | --- | --- |
| 1 | 18 | 4 | No |
| 2 | 18 | 4 | No |
| 3 | 19 | 4 | Yes |
| 4 | 19 | 4 | No |
| 5 | 18 | 3 | Yes |
| 6 | 19 | 3 | Yes |
| 7 | 18 | 2 | No |
| 8 | 18 | 2 | No |
| 9 | 20 | 2 | No |
| 10 | 19 | 2 | No |
| 11 | 21 | 2 | No |
| 12 | 19 | 1 | Yes |
| 13 | 18 | 1 | No |
| 14 | 19 | 1 | No |
| 15 | 20 | 1 | No |
| 16 | 23 | 1 | No |
| 17 | 20 | 1 | No |
| 18 | 24 | 1 | No |
| 19 | 19 | 1 | No |
| 20 | 20 | 1 | No |
| 21 | 18 | 1 | No |
| 22 | 18 | 1 | Yes |
| 23 | 19 | 1 | No |
| 24 | 21 | 1 | No |
| 25 | 19 | 1 | No |
| 26 | 18 | 1 | No |
| 27 | 21 | 1 | No |
| 28 | 19 | 1 | No |
| 29 | 20 | 1 | No |

*Note: Headache frequency was quantified by the question - “How frequently do you suffer from headaches?”; 1: less than once per month,  2: 1 - 2 per month, 3: 3 - 5 per month, 4: more than 5 per month.
